# Evaluation of rehabilitation exercise effects by using gradation-based skeletal muscle echo intensity in older individuals: a one-group before-and-after trial study

**DOI:** 10.1186/s12877-021-02423-3

**Published:** 2021-09-06

**Authors:** Akito Yoshiko, Takashi Kaji, Tsuyoshi Kozuka, Takayuki Sawazaki, Hiroshi Akima

**Affiliations:** 1 Faculty of Liberal Arts and Sciences, Chukyo University, Toyota, Aichi Japan; 2 Kajinoki Medical Clinic, Kani, Gifu, Japan; 3 Research Center of Health, Physical Fitness & Sports, Nagoya University, Nagoya, Aichi Japan; 4 Graduate School of Education and Human Development, Nagoya University, Nagoya, Aichi Japan

**Keywords:** Gradation-based echo intensity, Muscle thickness, Rehabilitation exercise, Elderly individuals

## Abstract

**Background:**

Higher muscle echo intensity (EI) reflects higher content of fat and/or connective tissue within skeletal muscle, eventually inducing lower muscle strength, physical dysfunction, and metabolic impairment. Continuous exercise decreases muscle EI in older individuals; however, it is not well understood how several months’ rehabilitation exercise affects gradation-based EI. The purpose of this study was to investigate the effects of 6 months of rehabilitation exercise on gradation-based higher and lower EI in older men and women.

**Methods:**

Twenty-seven men and women (7 men, 20 women; age, 75.6 ± 6.4 years; height, 154.3 ± 8.5 cm; weight, 55.8 ± 9.7 kg) participated in this study. This study was a one-group before-and-after trial. They needed long-term care for activities of daily living. They performed rehabilitation exercises consisting of resistance exercises using a hydraulic resistance machine, stretching, and aerobic exercises using a recumbent bicycle once or twice a week for 6 months. B-mode ultrasonographic transverse image was taken from thigh muscles, e.g., rectus femoris, vastus lateralis, and biceps femoris. We calculated gradation-based cross-sectional area (CSA) from thigh muscles by dividing 256 greyscale level to 10 different components levels (e.g., 0–24, 25–49, 50–74, …, 200–224 and 225–249 a.u.).

**Results:**

Lowest EI (e.g., 0–24 a.u.) CSA of thigh muscle was significantly increased after the exercise (0.3 ± 0.3 to 1.0 ± 0.8 cm^2^; *P* < 0.05). Middle to higher EI (e.g., 50–74, 75–99, 100–124, 125–149, 150–174, 175–199 and 200–224 a.u.) CSAs were significantly decreased from 23.0 to 68.7% after the exercise (*P* < 0.05).

**Conclusions:**

Several months’ rehabilitation exercise affected both lower and higher EI in older men and women. This result suggests that rehabilitation exercise changes muscle composition by increasing contractile muscle tissue and decreasing fat and connective tissues.

## Background

With advancing sarcopenia, muscle quality worsens because of the increased intramuscular adipose tissue (IntraMAT) and/or connective tissue within the muscle [1, 2]. Muscle quality, determined by the content of IntraMAT and/or connective tissue within the muscle, is negatively associated with locomotive functions and muscle glucose metabolism, eventually inducing a decrease in activities of daily living (ADL) and diabetes [3, 4]. Echo intensity (EI) of the skeletal muscle, as determined by ultrasonography, is one of the indicators of muscle quality [5, 6]. Many previous studies have shown the relationship between muscle EI and muscle strength, as well as daily physical function and daily physical activity [7–9]. Furthermore, EI in the thigh muscles of healthy older adults significantly decreased after 2.5–6.0 months of resistance training or walking intervention, implying an improvement in muscle quality by continuous exercise [10–12]. The effect of rehabilitation exercises on muscle size and physical functions, such as locomotive function, stair up and down, sit-to-stand, and knee extension strength, has been observed [13]; however, evidence of its effect on muscle quality remains unestablished. The intensity of traditional resistance and endurance training have been determined based on one-repetition maximum and ventilatory threshold [10, 14]; however, there have been challenges associated with measuring these parameters in older adults because of higher blood pressure, joint pain, and physical disability. Intensity is one of the key factors in the evaluation of the exercise effect; thus, it is speculated that the change in physiological muscle parameters in older individuals would be difficult to ascertain by low-intensity rehabilitation exercise.

Muscle EI is usually represented by grayscale levels. Many previous studies set the region of interest (ROI) on the muscle and measured the average EI from each pixel of ROI. Akima et al. [15] showed that the average EI in vastus lateralis (VL) and biceps femoris (BF) was related to the IntraMAT content (r = 0.40 to 0.49, *P* < 0.05) measured by magnetic resonance imaging (MRI), implying that higher average EI is positively correlated with higher IntraMAT content. More interestingly, the average EI significantly decreased after 1.5–6.0 months of resistance and endurance training [10–12]. Hypothetically, this decrease in average EI would be evoked by three type changes; 1) only decreased higher EI area of ROI, 2) only increased lower EI area of ROI, or 3) both decreased higher EI area and increased lower EI area of ROI. However, it is not well understood how several months of rehabilitation exercise affect higher and lower EI areas. Therefore, detecting these changes may be valuable for understanding the mechanism underlying the influence of rehabilitation exercise on physiological muscle parameters, including muscle cell hypertrophy and/or improvement in fat metabolism.

The purpose of this study was to investigate the effects of 6 months of rehabilitation exercise on gradation-based higher and lower muscle EI in older men and women. This may be the first study to clarify the change in muscle composition by using ultrasound images, and this concept was completely different from the previous study. We hypothesized that rehabilitation exercise decreased in the higher EI area but did not increase the lower EI area because the intensity of rehabilitation exercise was not enough to evoke muscle hypertrophy.

## Methods

### Participant characteristics and experimental procedure

This study was a one-group before-and-after trial. Twenty-seven older men and women requiring long-term care were enrolled (7 men, 20 women; age, 75.6 ± 6.4 years; height, 154.3 ± 8.5 cm; weight, 55.8 ± 9.7 kg; body mass index, 23.4 ± 2.9 kg/m^2^). We set two inclusion criteria: first, participants who fulfilled the requirements for long-term care set by the Ministry of Health, Labor and Welfare, Japan, and needed support for housework, medical care, and ADL for 25 to 70 min per day; second, age ≥ 65 years was included. The exclusion criteria were as follows: diagnosed impairment of cognitive function and dementia by a medical doctor; musculoskeletal, neuromuscular, orthopedic, and cardiovascular diseases; limited exercise, sports, and physical activities assessed by a medical doctor (T.K.). We enrolled participants who visited the clinic from 2018 to 2019. They had never participated in previous studies of ours [13]. Participants performed the 6 months’ rehabilitation exercises and were evaluated by ultrasound examination before and after the intervention. Before the experiment, the purpose, procedures, and risks associated with this study were explained to all participants, all of whom provided written informed consent to participate. All examination protocols were approved by the Institutional Review Board of the Research Center of Health, Physical Fitness and Sports at Nagoya University (No. 30–01) and the ethics committee of the Chukyo University (No. 2018–002) and were conducted in accordance with the ethical principles stated in the Declaration of Helsinki.

### Rehabilitation exercise program

As part of the rehabilitation program, participants performed rehabilitation exercises once or twice a week for 6 months. The exercise program consisted of resistance and endurance exercises under the supervision of a physical therapist (T.S.). This program was similarly performed in previous studies [13, 16]. Each session comprised of a warm-up (20 min), resistance tube, grasping a resistance ball with hands and catching a small ball using the toes (20 min), massage by a physical therapist (20 min), resistance exercise (20 min), endurance exercise (20 min), and a cool down (20 min), and was completed within 2.5 h, including a break. On each exercise day, participants performed resistance exercise programs using a hydraulic-resistance machine (Well-Round; Mizuno, Tokyo, Japan). Participants performed resistance exercises, such as hip adduction/abduction, knee extension/flexion, leg press, seated row, back extension/abdominal crunch, back twist, chest press, and shoulder press. Three or four resistance exercises were selected by the physical therapist considering the participant’s physical condition. Basically, we selected the programs based on the balance of upper and lower body parts to avoid excessive fatigue. Participants were instructed to move through the full range of motion for each resistance exercise movement as quickly as possible. Exercise intensity was basically set to approximately 30–50% of each participant’s maximum effort (same as 10–12 on the Borg scale) as previously performed [13, 16]. Each resistance exercise program continued for 5 min, with 2 min of rest between programs. After the short break, participants randomly performed endurance exercises on a recumbent stepper or recumbent cycle machine (NuStep, Ann Arbor, MI, USA). The workload was selectable from 1 (lowest) to 10 (highest); it was set as high as possible and continued for 10 min. All participants started with the lowest load (dial 1) in both resistance and endurance exercises in the first class, and the intensity was determined. The intensity of resistance and endurance exercises was set according to individuals’ exercise capacity level, and it was flexibly changed according to the participant’s physical condition on the day of the exercise. In some situations, such as when participants were not feeling well and/or had some pain, a physical therapist changed the exercise menu, volume, and intensity accordingly.

### Ultrasound measurements

**Fig. 1:**
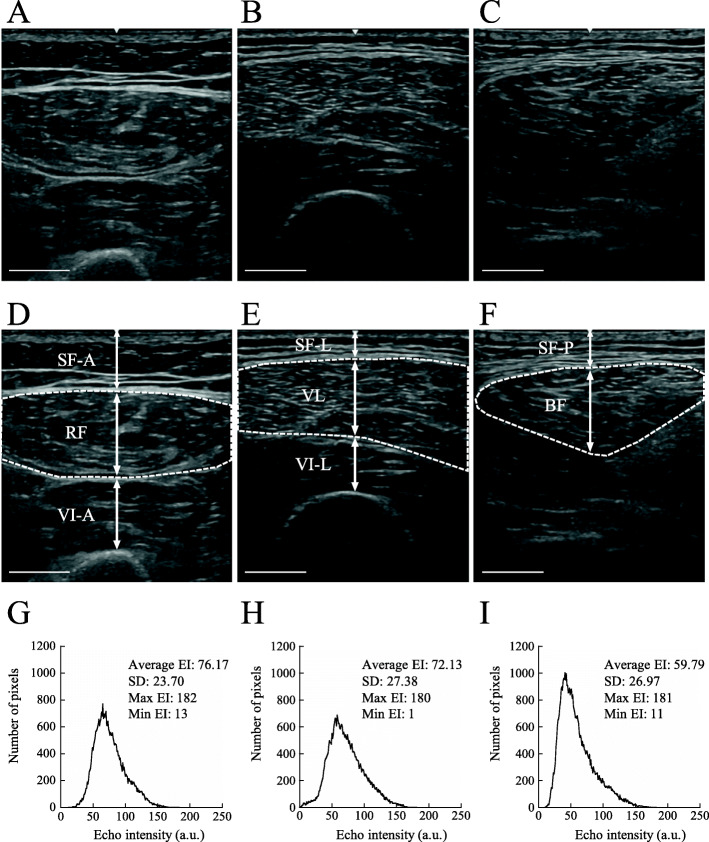
Representative ultrasound images in anterior, lateral, and posterior regions. **A** and **D** are the anterior, **B** and **E** are the lateral, and **C** and **F** are the posterior regions. Surface muscles (rectus femoris from **A**, vastus lateralis from **B** and biceps femoris from **C**) are traced using dotted lines in **D**, **E**, and **F**. Muscle echo intensities, and cross-sectional areas (CSAs) are measured from the dotted-line tracked region. Arrows show the thickness of the subcutaneous fat and muscle. **G**, **H**, and **I** is histograms of the ROI’s pixel intensity from rectus femoris (**G**), vastus lateralis (**H**) and biceps femoris (**I**). SF-A, subcutaneous fat thickness of the anterior region; RF, rectus femoris; VI-A, vastus intermedius of the anterior region; SF-L, subcutaneous fat thickness of the lateral region; VL, vastus lateralis; VI-L, vastus intermedius of the lateral region; SF-P, subcutaneous fat thickness of the posterior region; BF, biceps femoris. White bars are 1 cm in scale

Subcutaneous fat thickness, muscle thickness (MT), and EI of the mid-thigh were measured using an ultrasound device. Previous studies have described the procedure and technique of ultrasound measurement [7, 13, 16]. First, to avoid muscle contraction-induced blood flow and fluid shifts [17], participants rested in the supine position while fully extending the knee on a bed before the ultrasound measurements. After the 10-min rest period, we measured images from anterior and lateral regions (in spine position) and posterior regions (in prone position) with their knee joints fully extended (Fig. 1A, B and C). We measured the middle of the right thigh corresponding to the midpoint between the greater trochanter and lateral condyle. B-mode ultrasonographic device (LOGIQ e premium; GE Healthcare Japan, Tokyo, Japan) with a 3.8-cm width and 4.2- to 13.0-MHz linear array probe (L4-12t-RS; GE Healthcare Japan, Tokyo, Japan) was used to obtain images with the following acquisition parameters: frequency, 10 MHz; gain, 35 dB; depth, 3.0 to 5.0 cm; and focus point, 1 (top of the image). Depth was determined depending on the individual participant; it was generally ≤5.0 cm, and the same depth was used in each measurement. A water-soluble gel was applied to the scanning head of the probe to achieve acoustic coupling, and extra care was taken to avoid deformation of muscle morphology by placing the probe onto the skin surface. Three frozen images of each section were stored in DICOM format and transferred to a personal computer for later analysis.

ImageJ software, version 1.46 (National Institutes of Health, Bethesda, MD, USA), was used for analysis. Subcutaneous fat thickness was defined as the distance between the dermis and the lower boundary of the surface fascia. MT of the rectus femoris (RF), VL, and BF was defined as the distance between the inferior boundary of the surface fascia and the upper boundary of the deep fascia. In the case of the vastus intermedius (VI), MT was defined as the distance between the inferior border of the deep fascia and the superior border of the femur (i.e., deep aponeurosis) (Fig. 1D, E and F). The average MT in thigh muscles was calculated from that in RF, VL, VI and BF.

EI was assessed based on the pixel by pixel 256 grayscale level using ImageJ software and was expressed in arbitrary units (a.u.). We set the ROI by tracking the outline of the muscle as large as possible while excluding the visible fascia and the bone in the RF, VL, and BF (Fig. 1D, E and F). Histograms of the ROI’s pixel intensity from rectus femoris, vastus lateralis and biceps femoris were shown in Fig. 1G, H and I. The average EI inside the ROI and the cross-sectional area (CSA) of the ROI in three muscles were calculated for each image. The average EI and CSA in the thigh were calculated by averaging three muscles. The CSA was divided based on 10 gradation-based EIs (e.g., 0–24, 25–49, 50–74, 75–99, 100–124, 125–149, 150–174, 175–199, 200–224, and 225–249 a.u.). These procedures were performed before and after the 6 months of rehabilitation exercise.

### Statistical analysis

All values are reported as mean and standard deviation. The student paired *t*-test was used to compare before and after the exercise in subcutaneous fat thickness, MT, average EI, CSA of ROI, and gradation-based EI CSAs. Values of *P* < 0.05 were considered statistically significant. All statistical analyses were performed using IBM SPSS Statistics, version 22.0 J (IBM Japan, Tokyo, Japan).

## Results

Data on the subcutaneous fat thickness and MT are shown in Table 1, and average EI and CSA of ROI are shown in Table 2. MTs in RF, VI-anterior, VI-lateral, BF and thigh muscles were significantly increased after 6 months of exercise (*P* < 0.05). In the RF, VL, BF and thigh muscles, the average EI significantly decreased after the exercise compared with before the exercise (*P* < 0.05).

**Table 1:** Subcutaneous fat thickness and muscle thickness taken by ultrasonography at before and after the 6 months rehabilitation exercise

	Before (*n* = 27)			After (n = 27)		
*Subcutaneous fat thickness*						
Anterior (cm)	1.19	±	0.23	1.37	±	0.24
Lateral (cm)	0.62	±	0.29	0.59	±	0.28
Posterior (cm)	0.71	±	0.29	0.73	±	0.28
Thigh muscles (cm)	0.71	±	0.25	0.71	±	0.25
*Muscle thickness*						
Rectus femoris (cm)	1.14	±	0.27	1.23	±	0.28^*^
Vastus lateralis (cm)	1.31	±	0.35	1.37	±	0.34
Vastus intermedius anterior (cm)	0.99	±	0.29	1.09	±	0.35^*^
Vastus intermedius lateral (cm)	0.86	±	0.34	0.94	±	0.30^*^
Biceps femoris (cm)	1.67	±	0.34	2.00	±	0.38^*^
Thigh muscles (cm)	1.19	±	0.23	1.37	±	0.24^*^

Values are shown as mean ± SD

^*^P < 0.05 vs. Before

**Table 2:** Average echo intensity and cross-sectional area of the region of interest (ROI) at before and after the 6 months rehabilitation exercise

	Before (n = 27)			After (n = 27)			%change		
*Average echo intensity*									
Rectus femoris (a.u.)	4.47	±	1.09	4.39	±	0.89^*^	0.27	±	13.79
Vastus lateralis (a.u.)	66.99	±	14.97	56.08	±	17.28^*^	−16.85	±	15.70
Biceps femoris (a.u.)	59.12	±	13.24	42.04	±	13.43^*^	−28.43	±	21.01
Thigh muscles (a.u.)	67.20	±	10.59	52.71	±	13.10^*^	−22.04	±	12.57
*Cross-sectional area of ROI*									
Rectus femoris (cm^2^)	3.67	±	1.01	3.51	±	0.84	−1.19	±	20.37
Vastus lateralis (cm^2^)	4.89	±	1.73	4.68	±	1.39	−1.56	±	17.87
Biceps femoris (cm^2^)	4.86	±	1.41	4.99	±	1.32	8.99	±	35.12
Thigh muscles (cm^2^)	4.47	±	1.09	4.39	±	0.89	0.27	±	13.79

Values are shown as mean ± SD

^*^P < 0.05 vs. Before

**Fig. 2:**
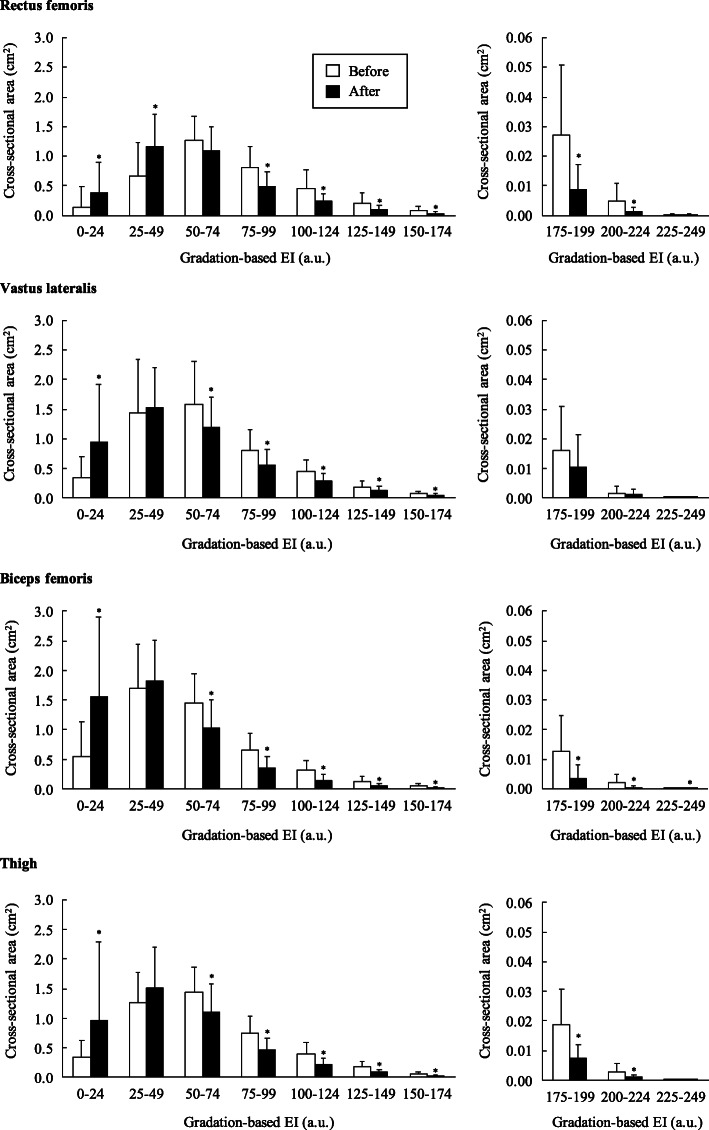
Gradation-based echo intensity (EI) cross-sectional areas before and after the 6 months rehabilitation exercise. * *P* < 0.05 vs. before

Gradation-based EI CSAs significantly increased at 0–24 a.u. in RF, VL, BF, and thigh (*P* < 0.05) and at 25–49 a.u. in RF (*P* < 0.05), whereas they significantly decreased at 50–74 a.u. in VL, BF, and thigh (*P* < 0.05), at 75–99 to 150–174 a.u. in RF, VL, BF, and thigh (*P* < 0.05), at 175–199 a.u. in RF, BF, and thigh, at 200–224 a.u. in RF, BF, and thigh (*P* < 0.05), and at 225–249 a.u. in BF (*P* < 0.05) (Fig. 2).

## Discussion

With aging, there is loss of skeletal muscle size in older individuals, eventually leading to a decline in force generation. This symptom, also known as sarcopenia, seriously affects ADL and QOL in older individuals. We observed that MT significantly increased after 6 months of rehabilitation exercise in older men and women (Table 1). Increase in type I and II muscle fibers area is induced by strength training, even in older individuals [18, 19]. Further, training-induced muscle hypertrophy is confirmed by morphological muscle parameters, such as MT and muscle CSA [10, 13, 20]. Although the intensity of our rehabilitation exercise was expected to be lower than that of traditional resistance training, partial muscle hypertrophy in older individuals would be induced after several months of rehabilitation exercise in this study. This result was closely related to the characteristics of the participants who fulfilled the requirements for long-term care and needed support for ADL includes routine housework and medical care. The baseline MTs of RF, VL, and VI in the participants were 20 to 30% lower compared with those of community-dwelling older individuals [7]. Chang et al. [21] showed that MT in RF in older Asian adults with lower muscle function was 1.30 ± 0.28 cm, and this rate was higher than that of our participants. Therefore, our participants have lower MT with a high possibility of sarcopenia at baseline. Muscle size is one of the important parameters to diagnose sarcopenia and it has been specially assessed as a factor relating to mobility function. Thus, the positive effects of rehabilitation exercise on MT observed in this study could significantly contribute to prevention and recovery from sarcopenia.

Muscle quality, evaluated by fat and/or connective tissue infiltration into skeletal muscle, was confirmed as a parameter of muscle composition. Decrease in average EI was demonstrated to be an indicator of muscle quality in several months of rehabilitation exercise (Table 2). Muscle quality has been demonstrated to worsen with aging [22], and lower muscle quality in the thigh (determined by a higher EI) indicated lower force and torque generation [8, 9] and lower locomotive function [23]. Many studies investigated the effect of continuous exercise on muscle quality in older individuals to improve muscle quality, and they reported that average muscle EI decreased after the resistance exercise [10, 11], concurrent resistance and endurance exercise [14], walking [12], and multiple rehabilitation exercise [13]. Interestingly, Radaelli et al. [10] investigated the effect of two-intensity resistance training on EI in RF. They reported that a significant decrease in EI after the 20 week-higher (three sets per exercise) and lower (one set per exercise) intensity training were observed alongside a greater change in higher intensity training (16.7 ± 8.6%) than in lower intensity training (8.7 ± 12.9%). The change in average EI in RF was 20.19 ± 13.37% in our study (Table 2), and that was higher than that of higher intensity training in the study by Radaelli et al. [10]. Our participants performed resistance and endurance exercise approximately 30–50% of each participant’s maximum effort (similar to 10–12 by Borg scale), and this level was one step before feeling the hardness (i.e., border intensity from light to somewhat hard). In contrast, Radaelli et al. (2014) investigated the effect of resistance training using the wight at 10 to 20 repetition maximum. Furthermore, Wilhelm et al. (2014) reported that EI significantly decreased after 12 weeks of endurance training with 85 to 95% heart rate equivalent to the second ventilatory threshold. Our exercise intensity was somehow low compared with these studies; however, we observed that the rehabilitation exercise definitely decreased the average EI in older individuals even at lower intensity. The rate of decreased average EI was dependent on factors, such as exercise type, exercise duration, frequency, and participant characteristics (age, sex, and ADL level). It would be necessary to determine these details considering the situation and participants’ condition; however, the contribution of these factors should be identified to establish an efficient exercise program in further studies.

To our knowledge, this is the first study to measure muscle EI by gradation-based CSA. Gradation-based EI CSAs significantly increased at lower intensity levels, such as 0–24 a.u., and decreased at middle and higher intensity levels, such as 50–74 to 200–224 a.u. (Fig. 2). This result showed that the numbers of darker pixels increased and that of brighter pixels decreased significantly after the rehabilitation exercise, respectively. In many previous studies, contractile tissue (muscle tissue) CSA and non-contractile tissue (fat and connective tissue) CSA have been measured by applying the difference in X-ray absorption rate in computed tomography (CT) imaging and relaxation time in MRI. Kent-Braun et al. [24] showed that older individuals had lower contractile tissue CSA and higher non-contractile tissue CSA compared with younger individuals in the tibialis anterior muscle. It has been confirmed that higher average EI is associated with higher fat and/or connective tissue concentration in muscle [1, 2]. Further, Akima et al. [15] showed the relationship between average EI and non-contractile tissue (e.g., IntraMAT) content determined by MRI (r = 0.40–0.49, *P* < 0.05). Although training affected the decrease in average EI [10, 11, 13, 14], the state of the muscle alongside the change in EI was unknown. We suggested that the change in gradation-based EI CSAs can potentially reveal the mechanism underlying the influence of rehabilitation exercise on contractile and non-contractile tissues. Contrary to our hypothesis, an increase of lower EI CSA and a decrease of higher EI CSA were observed (Fig. 2). These results suggest that the rehabilitation exercise induced an increase in the contractile tissue and a decrease in non-contractile tissue size, respectively; however, evidence on where the boundary of EI (to separate contractile and non-contractile tissue) lies remains unavailable. Further, the lower resolution of the ultrasound image would influence specifying tissue determination. The ultrasound image resolution was 80 × 80 μm (i.e., 6400 μm^2^) to 130 × 130 μm (16,900 μm^2^) per pixel. Considering that one muscle fiber is 4000 to 5300 μm^2^ in older individuals [25–27], one pixel contains one to three fibers. Whether gradation-based EI by ultrasound image can truly detect the contractile and non-contractile tissue should be confirmed in further studies.

Many studies showed the feature of muscle EI in older individuals by evaluating the relationship with functional performance and investigating the continuous exercise intervention [6, 8–10, 12, 13]. In addition, our new analysis using gradation-based EI would be helpful in understanding contractile and non-contractile tissue changes brought about by exercise intervention in older individuals (Fig. 2). Because our participants had a unique characteristic (i.e., not completely healthy and needing long-term care), we used an original exercise menu. These points made it difficult to generalize our findings; however, we suggested that our findings had the potential to promote the study of exercise effect on muscle EI. Recently, Nagae et al. [28] reported that muscle EI became the main predictor of hospital-associated complications in acute hospitalized older individuals. Furthermore, a few studies assessed muscle EI in orthopedics and neurosurgery patients [29, 30]. Therefore, muscle EI was applied for a practical and clinical scene and it had a potential as being one of the new features of skeletal muscle; however, EI was only used under the research level. The European Working Group on Sarcopenia in Older People, which is the organization that determines sarcopenia diagnosis cut-off value, recently proposed the use of muscle EI as a new tool for the assessment of muscle quality in older individuals [31]. However, it is still not established as the guideline and cut-off values of muscle quality as similar to muscle size and strength because of lack of evidence. The use of simple ultrasonographic muscle EI will have an exciting potential to understand healthy status in addition to muscle size and strength in older individuals. In addition, a cohort study is further needed to investigate whether muscle quality is associated with the quality of life, risk of fall and disease, and mortality for older individuals.

There are a few limitations. First, we did not have a control group. A previous study investigated physical training effects on EI by comparing responses of the training group against the control group [14]. In addition, we previously reported that muscle EI in RF and BF did not change through the 12-month and 24-month control periods [13, 16]. Data from a control period may emphasize the effects of the exercise intervention. However, we observed changes in EI with an obvious enhancement of physical activity. Second, we did not measure daily physical activity level and food intake because of difficult restrictions in the participant’s daily life. EI is related to daily steps and moderate activity time [23]; thus, it may become a key factor in assessing how much the rehabilitation exercise promotes daily physical activity. The amount of intake and dietary nutrition is strongly associated with IntraMAT [20]; however, it is still unknown how these factors affect the change in muscle EI by exercise. Third, we could not completely control and unify the exercise menu, volume, and intensity among participants. The physical condition and motivation of our participants were varied because they needed long-term care for daily living. We calculated the coefficient of variance (CV) from the percentage change of EI as supporting data. The values were 0.66 (RF), 0.93 (VL), 0.73 (BF), and 0.57 (thigh muscles), and they were similar to those from our previous study that controlled exercise menu and volume (CVs of percentage change of EI were 0.63 to 0.83) [12]. Therefore, the effect of the variable for exercise menu, volume, and intensity would be small. However, investigation regarding the effect of exercise type, volume, and intensity on muscle EI is necessary for further research because it is not completely understood.

## Conclusions

In conclusion, we found that six months of exercise decreased the average EI with increasing MT in several regions of the thigh muscle. Further, lower EI CSA increased, and middle and higher EI CSAs decreased after the exercise. These results might be induced by decreasing fat and/or connective tissues and increasing contractile muscle tissue, respectively. Simultaneously considering the participants’ condition and nursing care level with the present results, rehabilitation exercise may be suitable for developing quantitative and qualitative parameters in older individuals, which could eventually lead to needlessness for long-term care.

## Data Availability

The datasets used and/or analyzed during the current study are available from the corresponding author on reasonable request.

## Abbreviations

ADL: Activities of daily living
BF: Biceps femoris
CSA: Cross-sectional area
CT: Computed tomography
EI: Echo intensity
IntraMAT: Intramuscular adipose tissue
MRI: Magnetic resonance imaging
MT: Muscle thickness
RF: Rectus femoris
TUG: Timed up and go
VI: Vastus intermedius
VL: Vastus lateralis
